# Circular RNA circYPEL2: A Novel Biomarker in Cervical Cancer

**DOI:** 10.3390/genes13010038

**Published:** 2021-12-23

**Authors:** Xinyang Zhang, Siqi Yang, Wenbo Chen, Xin Dong, Rongyu Zhang, Haidong Ye, Xiangfei Mei, Huan Liu, Yu Fang, Chunjiang He

**Affiliations:** 1 School of Basic Medical Sciences, Wuhan University, Wuhan 430071, China; zhangxinyang@whu.edu.cn (X.Z.); smyangsiqi@163.com (S.Y.); cwb528@whu.edu.cn (W.C.); dongxin@whu.edu.cn (X.D.); 2020203010023@whu.edu.cn (X.M.); 2 College of Biomedicine and Health, Huazhong Agricultural University, Wuhan 430070, China; zry@webmail.hzau.edu.cn (R.Z.); 14779862806@163.com (H.Y.); che@whu.edu.cn (C.H.); 3 University of Science and Technology of China, Hefei 230026, China

**Keywords:** cervical cancer, circRNA, biomarker

## Abstract

Cervical cancer (CC) is one of the most threatening diseases in women. Circular RNAs (circRNAs) have been reported to be cancer hallmarks, but typical circRNAs in CC were rarely indicated. Through high-throughput sequencing in CC and normal cervix tissues, circYPEL2 (hsa_circ_0005600) was proposed as a candidate circRNA. CircYPEL2 exhibited significantly high expression in CC tissue and strong stability in CC cell lines. Furthermore, knockdown and overexpression of circYPEL2 indicated the potential involvement in CC proliferation, migration and invasion. Finally, the downstream regulatory genes of circYPEL2 were investigated by knockdown experiment in CC cell lines with high-throughput sequencing. In summary, our work identified circYPEL2 as a potential biomarker for clinical research of cervical cancer.

## 1. Introduction

Cervical cancer (CC) is the fourth most common cancer in women, causing death in up to 55% of patients. Human papillomavirus (HPV) infection is the major factor underlying CC [1]. More than 100 types of HPV have been identified as high-risk viruses based on their oncogenic characteristics, among which HPV 16 and HPV 18 are the most critical carcinogenic subtypes [2]. With the widespread application of vaccines against HPV and early cancer screening, an increasing number of patients can be treated at the early stages of the disease [3]. Nevertheless, the current method for CC screening is dependent on microscopic examination of cervical cells and HPV testing, which includes several complicated steps and is costly [4]. Identifying novel biomarkers for CC screening is a candidate procedure to improve the accuracy of diagnosis.

As a new family of noncoding RNAs, circular RNAs (circRNAs) are formed by back-splicing of exons/introns. Compared to linear transcripts, circRNAs do not contain a 5′-terminal cap structure or 3′-terminal poly A, rendering them more resistant to exonucleases and leading to relatively high stability in tissue and cells [5,6,7,8]. CircRNAs have been widely reported exerting functions as competing endogenous RNAs (ceRNAs). In addition, circRNAs can interact with RNA-binding proteins [9] and host DNA [10], and may translate functional proteins [11]. CircRNAs have been indicated as hallmarks in many cancers. For example, circFOXO3 can activate the expression of host gene FOXO3 by binding with p53 and MDM2, resulting in the increased apoptosis of breast cancer cells [12]. CircMTO1 was significantly downregulated in HCC and correlated with the survival of HCC patients [13]. CircTLK1 exacerbates renal cancer by activating CBX4 via competitive interaction with miR-136-5p [14]. CircSLC8A1 has been defined as a potential biomarker for the diagnosis of bladder cancer [15]. Though circRNAs have been revealed playing important roles in tumorigenesis, the function and regulation mechanism of circRNAs in CC are less known.

In this study, we performed expression profiling of circRNA in clinical CC and normal control samples using high-throughput sequencing and identified a CC-associated circRNA circYPEL2, which promoted the proliferation, invasion and migration of CC cells. Knockdown of circYPEL2 regulated downstream genes. Our work indicated that circYPEL2 may act as a potential biomarker in the development of CC.

## 2. Materials and Methods

### 2.1. Sample Treatment and Cell Culture

CC and normal control tissues were collected from CC patients and healthy volunteers from Wuhan University Zhongnan Hospital (Wuhan, China). Total RNA was extracted for whole transcriptome sequencing. RNA sequencing was performed using an Illumina HiSeq 2000, and all raw data were deposited in the Gene Expression Omnibus (GEO) repository (https://www.ncbi.nlm.nih.gov/geo/query/acc.cgi, accession number GSE173112). The CC cell lines HeLa and SiHa, and human normal cell line 293T were maintained in DMEM (HyClone, Los Angeles, CA, USA), supplemented with 10% fetal bovine serum (Gibco, Carlsbad, CA, USA) and 1% penicillin-streptomycin (Gibco, Carlsbad, CA, USA) and cultured at 37 °C in a humidified 5% CO_2_ incubator.

### 2.2. Processing of Sequencing Data

Low-quality reads and sequencing adapters were removed by Trim-Galore to obtain clean data. Then, the circular transcripts were identified and the circular/linear ratios (CLR) were calculated by CLEAR [16]. The linear transcripts were mapped to the hg19 genome by STAR [17] and counts were quantified using featureCounts [18]. TPM of linear transcripts were calculated based on mapped read counts [19,20]. Differential expression analysis of circRNA and mRNA was performed using the DESeq2 package [21], genes with |Log2Fold Change| > 1 and adjusted *p-*value < 0.05 were considered as differentially expressed. Pheatmap package was used for clustering analysis. GO enrichment analysis was performed using the clusterProfiler [22]. The interactions of the downstream genes of circRNA were obtained from STRING (https://cn.string-db.org/, accessed on 1 September 2021).

### 2.3. Total RNA and Genomic DNA Isolation

Total RNA was extracted using TRIzol reagent (Invitrogen, Carlsbad, CA, USA) according to the manufacturer’s instructions. The gDNA was extracted using a TIANamp Genomic DNA Kit (TIANGEN, Beijing, China).

### 2.4. RT-PCR and qPCR

Total RNA was reverse-transcribed using a Hifair^®^ Ⅱ 1st Strand cDNA Synthesis Kit (gDNA digester plus) (Yeasen, Shanghai, China) according to the manufacturer’s instructions. For PCR, 2 × Hieff™ PCR Master Mix (With Dye) (Yeasen, Shanghai, China) was used. The cDNA and gDNA PCR products were evaluated using 2% agarose gel electrophoresis. The qPCR was conducted using 2 × SYBR Green qPCR Master Mix (Bimake, Houston, TX, USA). GAPDH, β-actin and U1 were used as controls, and relative expression levels were calculated using the 2^−ΔΔCt^ formula. All primer sequences are listed in Supplementary Table S1.

### 2.5. RNase R and Actinomycin D Assay

The 2 μg RNA was incubated at 37 °C for 15 min with or without 3 U/μg RNase R (Lucigen, Ogden, UT, USA), and inactivated at 70 °C for 10 min, then analyzed by RT-PCR. HeLa and SiHa cells were incubated in DMEM with or without 2 μg/mL actinomycin D (MCE, Jersey, NJ, USA) for 24 h before RNA extraction [23,24]. The expression of circYPEL2 and YPEL2 mRNA was analyzed using RT-PCR.

### 2.6. Fractionation of Nuclear and Cytoplasma

Approximately 10^6^–10^7^ cells were collected, washed with ice-cold PBS twice and resuspended in 300 µL lysis buffer (1% NP-40, 0.5% sodium deoxycholate, 5 mM EDTA, 1 mM DTT, 1 mM PMSF, 2 mM VRC, 15% glycerol, 1 × proteinase inhibitor cocktail), incubated on ice for 5 min and centrifuged at 4000× *g* for 1 min at 4 °C, and the supernatant was saved for cytoplasmic fractionation. The precipitated nuclei were further washed once with lysis buffer, resuspended in 300 mL lysis buffer, sonicated and centrifuged at 13,000 rpm for 10 min at 4 °C. The supernatant was saved as the nuclear extract.

### 2.7. Plasmid Construction and Cell Transfection

For circYPEL2 overexpression, the sequence for exon 2 of YPEL2 was amplified using PrimerSTAR Max DNA Polymerase Mix (Takara, Dalian, China) and then inserted into pLCDH-ciR (Geenseed, Guangzhou, China), which was reconstructed by inserting a front circular frame and back circular frame to promote RNA circularization. Cells were transfected using Zlipo2000 (Zomanbio, Beijing, China) and PepMute (SignaGen, Baltimore, MD, USA). All primers and oligonucleotide sequences are listed in Supplementary Table S1.

### 2.8. Colony Formation Assay

HeLa and SiHa cells transfected with the plasmids or siRNA were cultured in 6-well plates at a density of 300–500 cells per well. Plates were incubated at 37 °C in 5% CO_2_ for 10–14 days, and colonies with more than 50 cells were scored. Cell colonies were immobilized with 4% paraformaldehyde (Biosharp, Hefei, China), stained with 0.5% crystal violet solution (Solarbio, Beijing, China), and then visualized under a microscope.

### 2.9. CCK-8 Assay

The proliferation of CC cells was determined using Cell Counting Kit-8 (CCK-8, Biosharp, Hefei, China) according to the manufacturer’s instructions. In brief, HeLa and SiHa cells were seeded into 96-well plates at a density of 2000 cells/well after transfection. Then, 10 μL CCK-8 solution was added to each well after 24, 48 and 72 h. The absorbance was measured at a wavelength of 450 nm by a Multiskan FC Microplate Photometer (Thermo Fisher, Waltham, MA, USA).

### 2.10. Cell Migration and Invasion Assay

Transwell assays were used to evaluate the invasion and migration capacities of CC cells in vitro. For cell migration, HeLa and SiHa cells were harvested after transfection for 24 h. A total of 2 × 10^4^ cells were seeded in the upper chamber with 500 μL serum-free medium, and medium containing 10% FBS was added into the lower chamber as a chemoattractant. For the cell invasion assay, 100 µL Matrigel was added to the upper chamber. After incubation for 24 h, cells on the upper surface of the membrane were removed by wiping with a Q-tip, and the invaded or migrated cells were immobilized with 4% paraformaldehyde (Biosharp, Hefei, China) and stained with 0.5% crystal violet solution (Solarbio, Beijing, China). Then, positive cells were quantified.

### 2.11. Statistical Analysis

For qPCR, colony formation assay, cell migration and invasion assay, all these experiments were independently conducted three times. Relative expression or cell number were presented as the mean ± SD. Statistical analyses were performed using GraphPad Prism 7.0 (La Jolla, San Diego, CA, USA). The Student unpaired *t*-test was performed to identify the differences between two experimental groups. For CCK-8 assay, experiments were independently conducted three times and six technical repetitions were performed at each time point. OD450 values were presented as the mean ± SD. Two-way ANOVA was performed to identify the effect of two factors on cell growth and multiple comparison adjustment was performed by Benjamin–Hochberg algorithm for the comparison at each point time. Statistical analyses were performed using GraphPad Prism 7.0 (La Jolla, San Diego, CA, USA) and R (www.r-project.org, accessed on 4 September 2021). As statistical significance, * *p* < 0.05, ** *p* < 0.01, *** *p* < 0.001 and **** *p* < 0.0001 were considered.

## 3. Results

### 3.1. Identification of CC-Associated circRNAs

**Figure 1:**
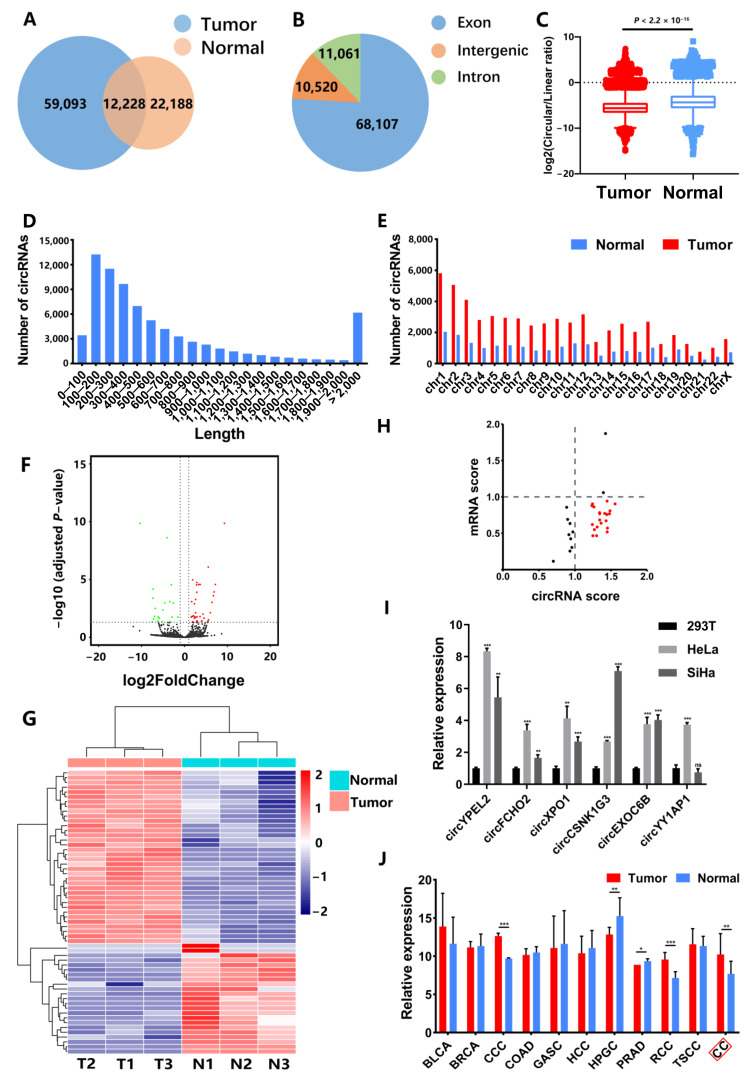
Identification of circRNA expression in CC. (**A**) Number of circRNAs detected in tumor (3 samples) and normal tissues (3 samples). (**B**) The gene location of circRNAs. (**C**) The ratios of circular and linear RNA expression in tumor (3 samples) and normal tissues (3 samples) (*p-*value < 2.2 × 10^−16^). (**D**) The length distribution of circRNAs. (**E**) The chromosome locations of circRNAs. (**F**) Volcano plot of differentially expressed circRNAs in CC. Red and green points represent significantly upregulated and downregulated circRNAs, respectively (|log2FoldChange| > 1, adjusted *p-*value < 0.05). (**G**) Clustering analysis of circRNAs with significantly different expression between tumor and normal samples. Normalized counts from DESeq2 were used for depicting the heatmap. (**H**) The anchor of circYPEL2. Points represent differentially expressed circRNAs (|log2FoldChange| > 1, adjusted *p-*value < 0.05, average expression > 25). Red points represent circRNAs that were upregulated in tumors, while their host genes were downregulated. (**I**) RT-PCR of differentially expressed circRNA in HeLa and SiHa cell lines. (**J**) Expression of circYPEL2 in various cancers based on CSCD2 database. (Mean ± SD of three experimental replicates are presented. * Represents *p* < 0.05, ** represents *p* < 0.01, *** represents *p* < 0.001).

We performed RNA-seq for three CC tissues and three normal cervical samples and identified circRNAs. Results indicated a total of 93,509 circRNAs were detected in CC and normal samples. Overlap analysis revealed 59,093 circRNAs are only expressed in CC and 22,188 circRNAs are only expressed in normal samples (Figure 1A). In total circRNAs, 68,107 (76%) circRNAs were derived from the exonic type, while 11,060 (12%) and 10,520 (12%) circRNAs were derived from intronic and intergenic regions, respectively (Figure 1B). In addition, we calculated the ratio of circular to linear transcripts (CLR) in tumor and normal tissues. Results showed that CLR in CC tissues was significantly lower than that in normal controls, which was identical to previous works [8,25], revealing an average lower expression of circRNAs in cancers (Figure 1C).

The length of circRNA transcripts were mostly from 100 bp to 700 bp (Figure 1D). In addition, the chromosomal distribution of circRNA transcripts exhibited that there were more circRNAs in CC than normal samples in all chromosomes (Figure 1E). Location analysis exhibited most of circRNAs were derived from encoded genes (Supplementary Figure S1A).

A total of 59 circRNAs were detected with significantly differential expression between CC and normal samples (|log2FoldChange| > 1 and adjusted *p*-value < 0.05). Among them, 36 circRNAs were upregulated and 23 circRNAs were downregulated in tumor tissues (Figure 1F,G and Supplementary Figure S1B). Notably, we observed 65.6% highly expressed circRNAs derived from lowly expressed host genes in CC (Figure 1H, red points). To verify the RNA-seq results, qPCR for these circRNAs were conducted in HeLa and SiHa cells, with 293T cell line, which is a widely used normal control according to previous works [26,27,28,29]. Results showed the expression of circYPEL2 was highest in seven circRNAs, which were consistent to RNA-seq results (Fold-change > 6, and adjusted *p*-value = 0.0090, Student unpaired *t*-test) (Figure 1I and Supplementary Figure S1D).

Next, we detected the expression of circYPEL2 in CSCD2 database [30], which includes circRNA expression in most of cancer types, and observed circYPEL2 is differentially expressed in cervical cancer (CC), as well as several other malignancies, including clear cell carcinoma (CCC), hypopharyngeal cancer (HPGC), prostate cancer (PRAD) and renal cell carcinoma (RCC) (Figure 1J), suggesting that circYPEL2 may play an important role in the development of cancers.

### 3.2. CircRNA Exerts Strong Stability in CC Cell Lines

**Figure 2:**
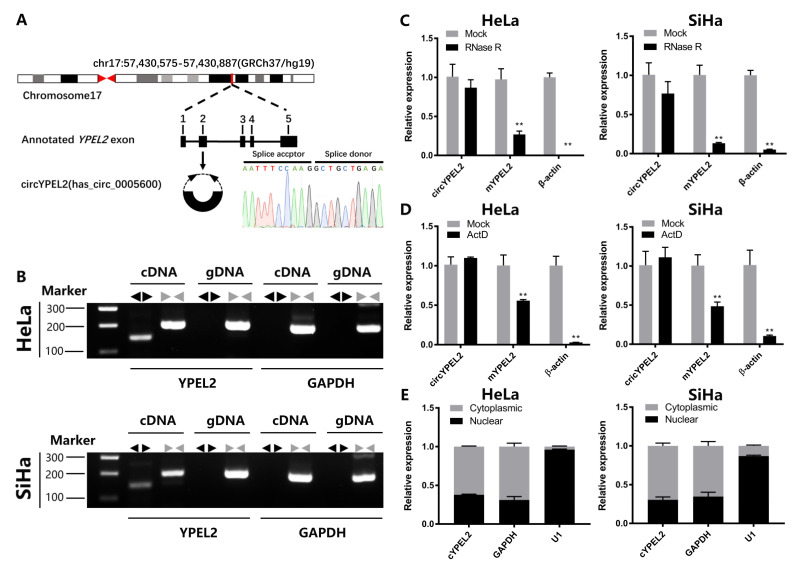
CircYPEL2 exhibits stability in CC cell lines. (**A**) Structures of the YPEL2 genome and transcript. CircYPEL2 is derived from exon 2. The back-splice junction of circYPEL2 was identified by Sanger sequencing. (**B**) RT-PCR assay with divergent or convergent primers indicated the expression of circYPEL2 in HeLa and SiHa cell lines. GAPDH was used as negative control. The cDNA: complementary DNA; gDNA: genomic DNA. (**C**) The qRT-PCR analysis of the expression of circYPEL2 and YPEL2 mRNA after treatment with RNase R in HeLa and SiHa cell lines. (**D**) The qRT-PCR analysis of the expression of circYPEL2 and YPEL2 mRNA in response to treatment with actinomycin D for 24 h in HeLa and SiHa cell lines. (**E**) Fractionation experiments of cytoplasmic and nuclear mRNA showed that circYPEL2 localized in the nucleus and cytoplasm. β-actin and U1 were applied as positive controls in the cytoplasm and nucleus, respectively. (Mean ± SD of three experimental replicates are presented. ** Represents *p* < 0.01).

CircYPEL2 is derived from exon 2 (312 bp) of YPEL2. The back-spliced junction site of circYPEL2 was amplified using divergent primers and confirmed by Sanger sequencing (Figure 2A). PCR using convergent primers and divergent primers amplifying linear and circular RNA was conducted in cDNA and genomic DNA (gDNA), respectively in HeLa and SiHa cells, and circYPEL2 was only amplified by divergent primers in cDNA but not in gDNA (Figure 2B). In addition, circYPEL2 exhibited resistance while the linear transcript exhibited significant degradation after treatment by RNase R (Figure 2C). Next, HeLa and SiHa cells were pretreated with actinomycin D, a transcription inhibitor, for 24 h, followed by qRT-PCR analysis. Comparing to the linear transcript, circYPEL2 exhibited strong resistance to actinomycin D, indicating distinct biological stability (Figure 2D). In addition, fractionation experiment of nuclear and cytoplasmic revealed that circYPEL2 was prominently localized in the cytoplasm rather than the nucleus (Figure 2E). These results revealed the features of circYPEL2 in CC cells and suggested a potential stable regulator in tumorigenesis.

### 3.3. Knockdown of circYPEL2 Attenuates the Proliferation, Migration and Invasion of CC

**Figure 3:**
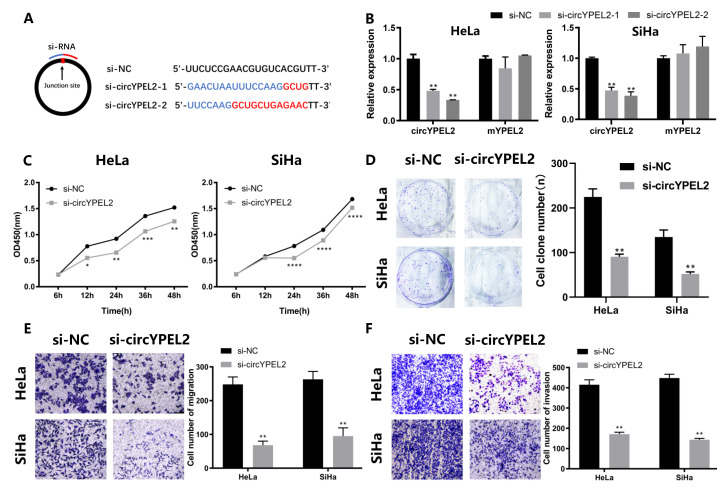
Knockdown of circYPEL2 suppresses cell proliferation, migration and invasion. (**A**) Schematic illustration and sequences of si-NC and si-circYPEL2. (**B**) Expression levels of circYPEL2 and YPEL2 mRNA in HeLa and SiHa cell lines transfected with si-circYPEL2. Si-NC was the negative control. (**C**) CCK-8 assays in HeLa and SiHa cell lines to detect cell viability at 0, 6, 12, 24, 36 and 48 h. (**D**) Clone formation assays in HeLa and SiHa cell lines to detect cell proliferation. (**E**,**F**) Transwell assay for cell migration and invasion ability in HeLa and SiHa cells. (Mean ± SD of three experimental replicates are presented. * Represents *p* < 0.05, ** represents *p* < 0.01, *** represents *p* < 0.001, **** represents *p* < 0.0001).

To investigate the regulatory mechanism of circYPEL2 in CC, transfection of short interfering RNAs (si-NC, si-circYPEL2-1, si-circYPEL2-2) was performed (Figure 3A). Si-circYPEL2-2 was selected for subsequent experiments due to its increased efficiency and specificity of interference (Figure 3B). The results of CCK-8 and colony formation assays indicated that the viability and proliferation of CC cells were inhibited by circYPEL2 knockdown (Figure 3C,D). Furthermore, transwell assays revealed significant abatement of cell migration and invasion upon circYPEL2 silencing (Figure 3E,F). In summary, knockdown of circYPEL2 attenuated the proliferation, migration and invasion of CC.

### 3.4. Overexpression of circYPEL2 Promotes the Proliferation, Migration and Invasion of CC

**Figure 4:**
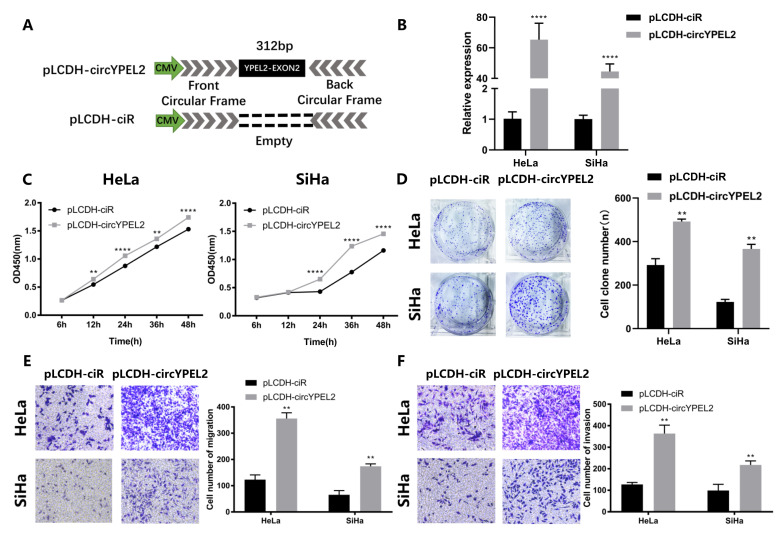
Overexpression of circYPEL2 promotes cell proliferation, migration and invasion. (**A**) Schematic illustration of the expression vector of circYPEL2. (**B**) Expression levels of circYPEL2 and YPEL2 mRNA in HeLa and SiHa cell lines transfected with the pLCDHircYPEL2 vector. The pLCDH-ciR vector was the negative control. (**C**) CCK-8 assays in HeLa and SiHa cell lines to detect cell viability at 0, 6, 12, 24, 36 and 48 h. (**D**) Clone formation assays in HeLa and SiHa cell lines to detect cell proliferation. (**E**,**F**) Transwell assay for cell migration and invasion ability in HeLa and SiHa cells. (Mean ± SD of three experimental replicates are presented. ** Represents *p* < 0.01, **** represents *p* < 0.0001).

To further confirm the function of circYPEL2, we constructed a circYPEL2 overexpression plasmid using the pLCDH-ciR vector (Figure 4A), which contains a front and back circular frame. The qRT-PCR analysis was then performed to ensure overexpression efficiency (Figure 4B). Next, the viability and proliferation of CC cells were evaluated. Cell proliferation was activated by pLCDH-circYPEL2 transfection (Figure 4C,D), and cell migration and invasion ability were induced as measured by transwell assay. Exogenous supplementation with circYPEL2 obviously contributed to migration and invasion (Figure 4E,F). These results further indicated that circYPEL2 promoted the progression of CC.

### 3.5. CircYPEL2 May Regulate CC via Downstream Genes

**Figure 5:**
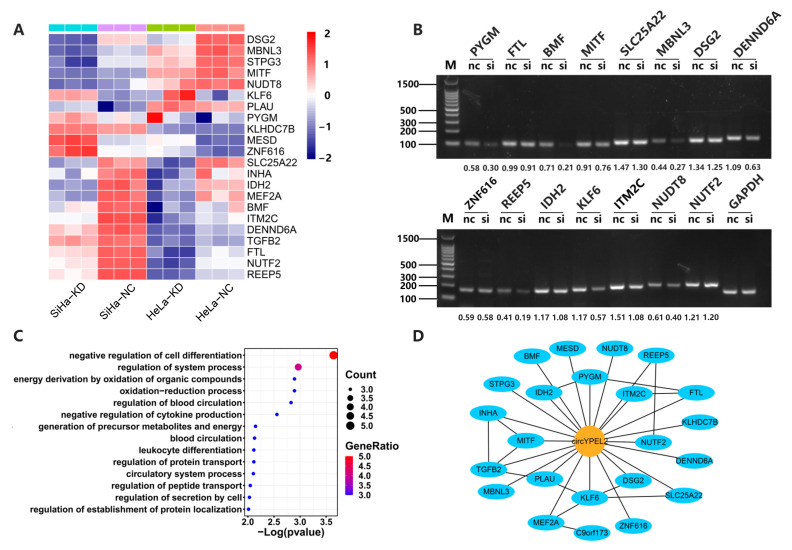
CircYPEL2 may regulate CC via downstream gene. (**A**) Clustering analysis of genes with significantly different expression in two cell lines treated with si-NC and si-circYPEL2 (|log2FoldChange| > 1, adjusted *p-*value < 0.05). TPM of genes were used for depicting the heatmap. (**B**) RT-PCR validation of differentially expressed genes. The nc represents si-NC and si represents si-circYPEL2. (**C**) Gene Ontology analysis of differentially expressed genes. (**D**) Regulatory network of circRNAs and coding genes. The blue and yellow nodes represent coding genes and circRNA, respectively.

To explore the potential regulatory mechanism of circYPEL2 in CC, we performed RNA-seq in HeLa and SiHa cell lines with knockdown of circYPEL2. Differential expression analysis revealed that 22 genes were significantly regulated by circYPEL2 knockdown in two cells (Figure 5A). RT-PCR was used to validate the significantly differentially expressed genes (Figure 5B). Gene Ontology analysis indicated that most of the differentially expressed genes were enriched in leukocyte differentiation, blood circulation and cell differentiation, which are correlated with proliferation and migration (Figure 5C). In addition, we observed these genes are enriched in GSEA hallmark pathways (https://www.gsea-msigdb.org/gsea/index.jsp, accessed on 15 September 2021), such as epithelial–mesenchymal transition (EMT) and KRAS signaling, as well as oxidative phosphorylation, hypoxia and reactive oxygen pathways, which are associated with tumorigenesis and energy metabolism [31,32,33] (Supplementary Table S2). Furthermore, the interaction network between these downstream genes of circYPEL2 was predicted (Figure 5D).

## 4. Discussion

As a new type of endogenous noncoding RNA, circRNAs are produced by precursor mRNA (premRNA) and have special structural stability [34]. The dysregulation of circRNAs in cancers has been widely reported, and accumulating evidence suggests that circRNAs are associated with tumor progression [30,35] and circRNAs are potential biomarkers for tumor diagnosis, treatment and prognosis [36,37]. In this study, high throughput sequencing revealed a total of 59 differentially expressed circRNAs between CC and normal controls. We analyzed expression of these circRNAs and found that highly expressed circRNAs were concomitant with low expression of host genes, such as circYPEL2 and circFCHO2, indicating an independent expression pattern of circRNA, which was also revealed in previous work [38]. In addition, circRNAs were more stable than their host genes in CC cells (Figure 2C). These results suggest that circRNAs are potential robust biomarker than linear genes.

Gain and loss of function experiments suggested that circYPEL2 may act as an oncogene in CC by regulating cell proliferation, invasion and migration. We further identified the downstream genes of circYPEL2 by performing high-throughput sequencing on CC cell lines with knockdown of circYPEL2. Genes such as PYGM, BMF, MBNL3, DENND6A, REEP5, KLF6 and ITM2C are potentially regulated by circYPEL2 and involved in cancer-specific pathways [39,40,41], which are needed to be validated in further studies.

## 5. Conclusions

In summary, combining with high throughput sequencing and experiment analysis, we explored the CC-associated circRNAs and investigated the potential function of circYPEL2 in CC cells, which provided a novel view for understanding the development of CC and diagnosis.

## Data Availability

All raw data were deposited in the Gene Expression Omnibus (GEO) repository (https://www.ncbi.nlm.nih.gov/geo/query/acc.cgi, accession number GSE173112).

## Supplementary Materials

The following are available online at https://www.mdpi.com/article/10.3390/genes13010038/s1, Figure S1: CircYPEL2 is upregulated in cervical cancer, Table S1: Primers used in qRT-PCR & RT-PCR analysis, Table S2: Differentially expressed genes enriched in GSEA hallmark pathways.
