# Supplementary material for: Circular RNA circYPEL2: A Novel Biomarker in Cervical Cancer
Source: Genes (Basel). 2021 Dec 23;13(1):38. doi: 10.3390/genes13010038 (PMC8774301; doi:10.3390/genes13010038)
Supplement: Supplementary file 1 [file genes-13-00038-s001.zip › Table S1.pdf]

Table S1. Primers used in qRT-PCR & RT-PCR analysis

Name	Squence(5'-3')	
	Forward	Reverse
circYPEL2	GCATTCACTGCAGAGCTCAC	TAAAGGCCGTGGTTTGGCA
circFCHO2	CTTCACCAGCAATCCAACTCC	TCCTGATTTCCAAGGCTGACA
circXPO1	TGCAACTGACACAGCTAGGC	GAGCTGCATGGTCTGCTAAC
circCSNK1G3	ATGTGGCAATTAAGTTGGAGCC	GAGCATGTTCATCCCATTCGT
circYY1AP1	TTCAGCCAAACAGCAGAAGGA	TAGGTTGGCCAGTAGTTCCTCA
mYPEL2	CCAGAGTTCACCCCACACTC	GCCCACAGCCCACATTAACTA
gYPEL2	CCGAAGACATCACCAGTGT	GGCAGATATGCCTGGAAAGT
mPYGM	TGGCCAAAGTGAAGCAGGAA	CGCTTCACCTGGATGTCGAA
mFTL	CAGCCTGGTCAATTTGTACCT	GCCAATTCGCGGAAGAAGTG
mBMF	TTTATGGCAATGCTGGCTATCG	GCAATCTGTACCTCTGCTTGATG
mMITF	CGACAGAAGAAACTGGAGCAC	AAATCTGGAGAGCAGAGACCC
mSLC25A22	GCCAGCCAAGCTCATCAATG	GAGGCAGTCGGACATGCTC
mMBNL3	TCCTGGAAACCCACCTCTT	CATCAGTAGGGTGAGCATAG
mDSG2	CTCAGGTGTGCAGCCTACTC	GTGGTGTTCCTAGCCGTCAT
mDENNE6A	GGGAGCCCCTTGTGGTTATG	GGAGCTTGGGTACGGGTAGT
mZNF616	GTGATCTGGGGATCGCTACG	TCAAATGCCCCTGAGTAGCC
mREEP5	CATCGCTCTTGGTGTCATC	CATCGCTCTTGGTGTCATC
mIDH2	CTGGCCACCCAGAAGTACAG	GGACTAGGCGTGGGATGTTT
mKLF6	GGCCAAGTTTACCTCCGACC	TAAGGCTTTTCTCCTGGCTTCC
mITMIC	CGAGAACTACGAGCGCATCA	AGGTAGGTCCCCCTCTTCAC
mNUDT8	GTTTCCCAGGCGGCAAGT	ACCTCATCTACCTCCTCCGAGTT
mNUTF2	ATGCCTTACGTGGGAAGGAC	ACCTCATCTACCTCCTCCGAGTT
GAPDH	GCACCACCAACTGCTTA	AGTAGAGGCAGGGATGAT
diverGAPDH	TTGCCCTCAACGACCACTTT	ACCAAATCCGTTGACTCCGA
β-actin	AGAGCTACGAGCTGCCTGAC	AGCACTGTGTTGGCGTACAG
RNU1	ACTTACCTGGCAGGGGAGATACC	CCACTACCACAAATTATGCAGTCG
RNU6	CTCGCTTCGGCAGCACA	AACGCTTCACGAATTTGCGT

Primer sequence for pLCDH-circYPEL2 vector construction	
Name	Squence(5'-3')
OE-circYPEL2-F	CGGAATTCTGAAATATGCTATCTTACAGGCTGCTGAGAACTAGCC
OE-circYPEL2-R	CGGGATCCTCAAGAAAAAATATATTCACCTTGGAAATTAGTTCATCATGAT
