# Supplementary material for: Circular RNA circYPEL2: A Novel Biomarker in Cervical Cancer
Source: Genes (Basel). 2021 Dec 23;13(1):38. doi: 10.3390/genes13010038 (PMC8774301; doi:10.3390/genes13010038)
Supplement: Supplementary file 1 [file genes-13-00038-s001.zip › Table S2.pdf]

Table S2. Differentially expressed genes enriched in GSEA hallmark pathways

GSEA Hallmark pathways	DEGs interacted with circYPEL2			
HALLMARK_TNFA_SIGNALING_VIA_NFKB	KLF6	PLAU		
HALLMARK_HYPOXIA	KLF6	PLAU	PYGM	INHA
HALLMARK_INFLAMMATORY_RESPONSE	KLF6	PLAU		
HALLMARK_IL2_STAT5_SIGNALING	KLF6			
HALLMARK_CHOLESTEROL_HOMEOSTASIS	PLAU			
HALLMARK_APICAL_SURFACE	PLAU			
HALLMARK_COMPLEMENT	PLAU			
HALLMARK_EPITHELIAL_MESENCHYMAL_TRANSITION	PLAU			
HALLMARK_COAGULATION	PLAU			
HALLMARK_KRAS_SIGNALING_UP	PLAU			
HALLMARK_MYOGENESIS	PYGM	MEF2A		
HALLMARK_ESTROGEN_RESPONSE_LATE	IDH2			
HALLMARK_OXIDATIVE_PHOSPHORYLATION	IDH2			
HALLMARK_BILE_ACID_METABOLISM	IDH2			
HALLMARK_PEROXISOME	IDH2			
HALLMARK_APOPTOSIS	BMF	TGFB2		
HALLMARK_XENOBIOTIC_METABOLISM	TGFB2			
HALLMARK_ALLOGRAFT_REJECTION	TGFB2			
HALLMARK_KRAS_SIGNALING_DN	TGFB2			
HALLMARK_REACTIVE_OXYGEN_SPECIES_PATHWAY	FTL			
HALLMARK_ADIPOGENESIS	REEP5			
